# Supplementary material for: Clonal hematopoiesis is associated with risk of severe Covid-19
Source: Nat Commun. 2021 Oct 13;12:5975. doi: 10.1038/s41467-021-26138-6 (PMC8514469; doi:10.1038/s41467-021-26138-6)
Supplement: Supplementary file 1 — Supplementary Information [file 41467_2021_26138_MOESM1_ESM.pdf]

Supplementary Information for “Clonal hematopoiesis is associated with risk of severe Covid-19”

SUPPLEMENTARY FIGURES

Supplementary Figure 1. Patient population by Covid-19 severity and CH driver status. ... 3
Supplementary Figure 2. Association between CH mutation type and Covid-19 severity ... 4
Supplementary Figure 3. Frequency of genes with non-driver mutations among individuals with severe Covid-19. ... 5
Supplementary Figure 4. Association between CH and Covid-19 severity stratified by the number of mutations ... 6
Supplementary Figure 5. Association between maximum VAF of CH-mutation(s) and Covid-19 severity ... 7
Supplementary Figure 6. Association between CH and non-invasive/invasive ventilation related to Covid-19 ... 8
Supplementary Figure 7. Frequency of CH mutation by genes and Covid-19 infection and severity status ... 9
Supplementary Figure 8. Proportion of individuals with CH by Covid-19 severity, testing status and age group ... 10
Supplementary Figure 9. Proportion of individuals with CH by Covid-19 severity and the most common primary tumor sites. ... 11
Supplementary Figure 10. Number of lines of antibiotics by the presence of clonal hematopoiesis and the development of clostridium difficile (C. diff) infection ... 12

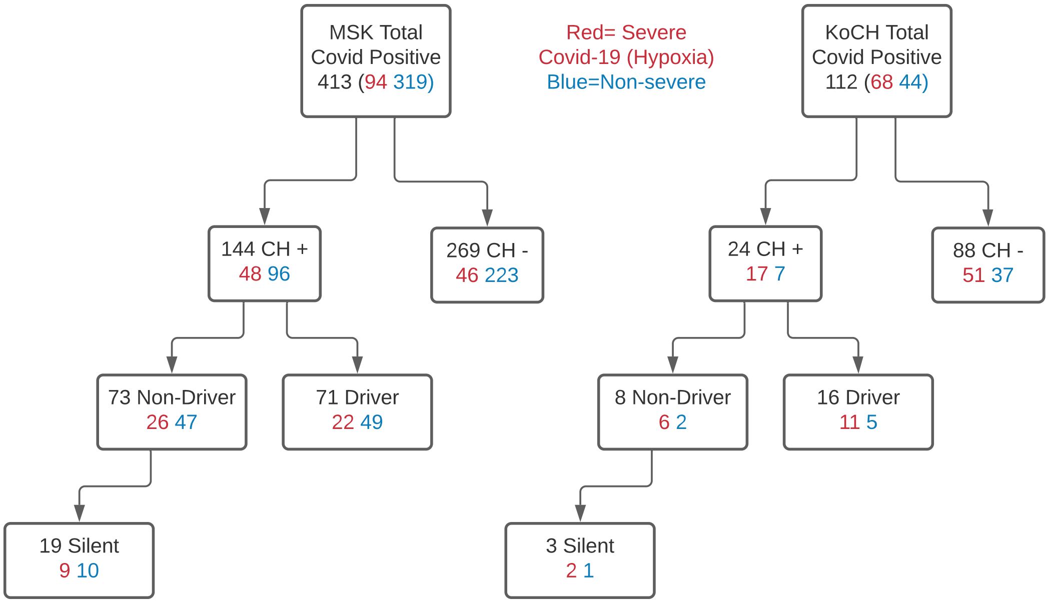

Supplementary Figure 1. Patient population by Covid-19 severity and CH driver status.

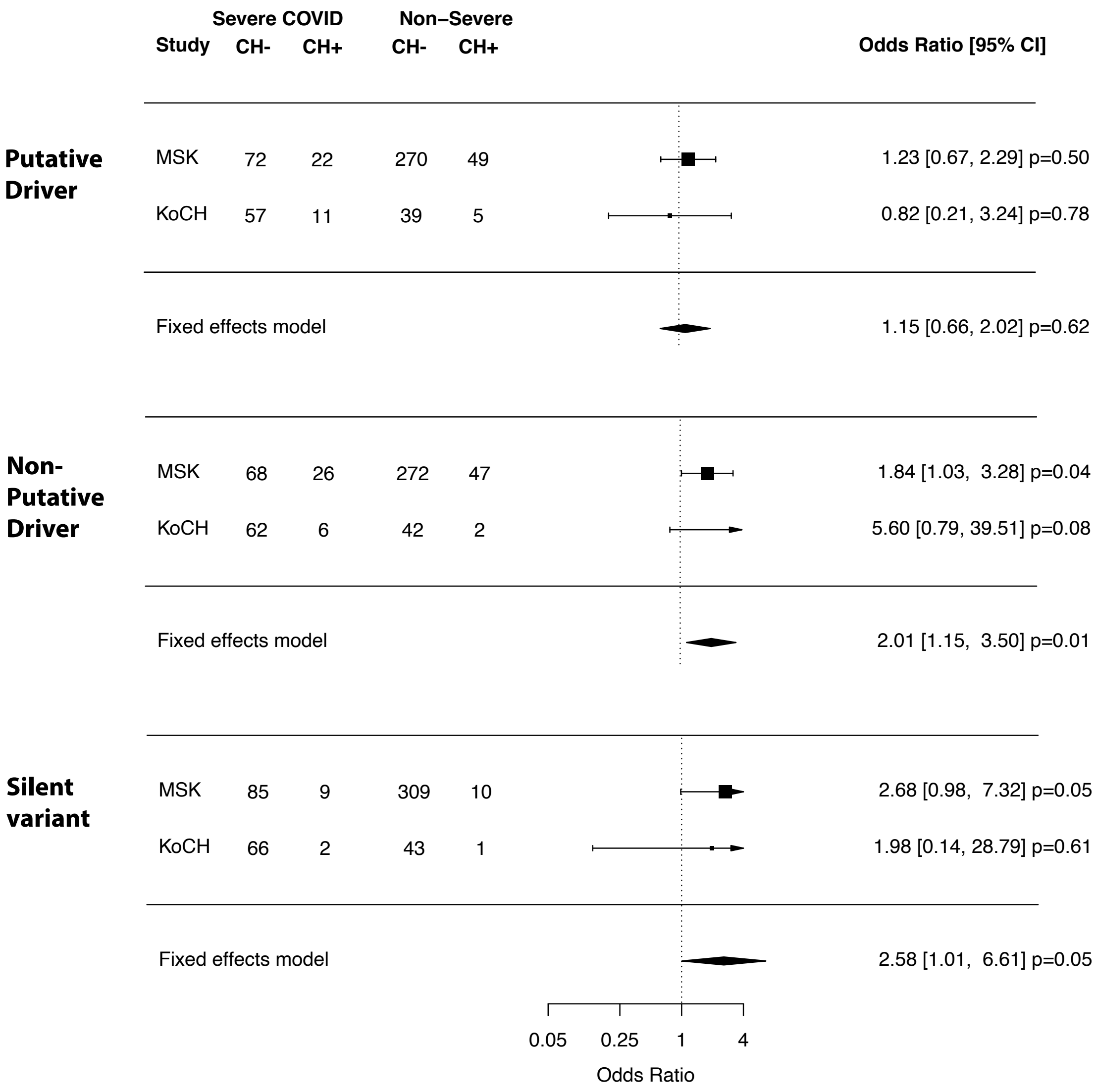

Supplementary Figure 2. **Association between CH mutation type and Covid-19 severity.** Shown are the odds ratio, 95% confidence interval and p-value (not adjusted for multiple hypothesis testing) from logistic regression adjusted for age, gender, race, smoking, diabetes, cardiovascular disease, COPD/asthma, BMI and month of Covid-19 diagnosis in 525 individuals. The MSK cohort was also adjusted for cancer primary site, exposure to cytotoxic cancer therapy before and after blood draw. Summary statistics for a fixed effects meta-analysis are shown.

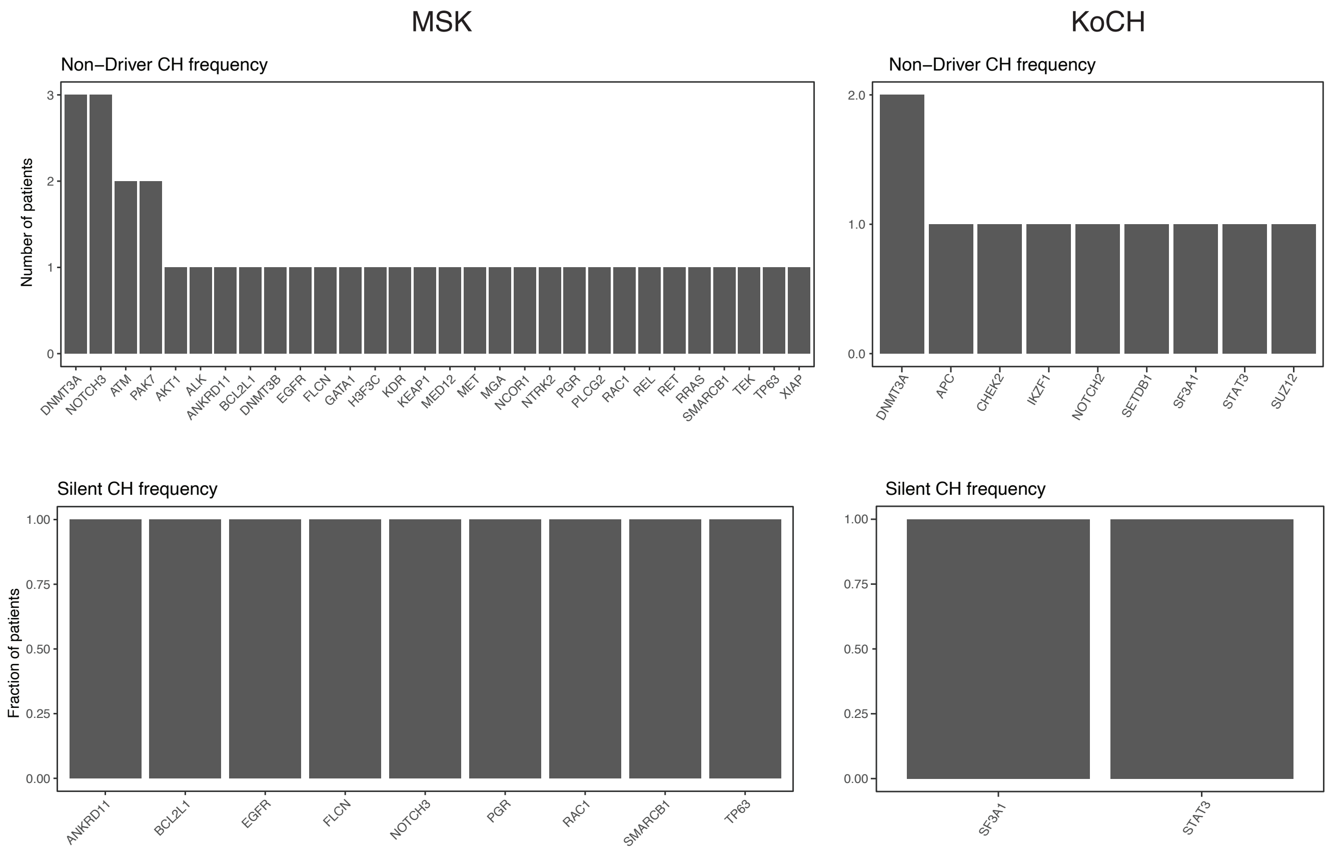

Supplementary Figure 3. Frequency of genes with non-driver mutations among individuals with severe Covid-19.

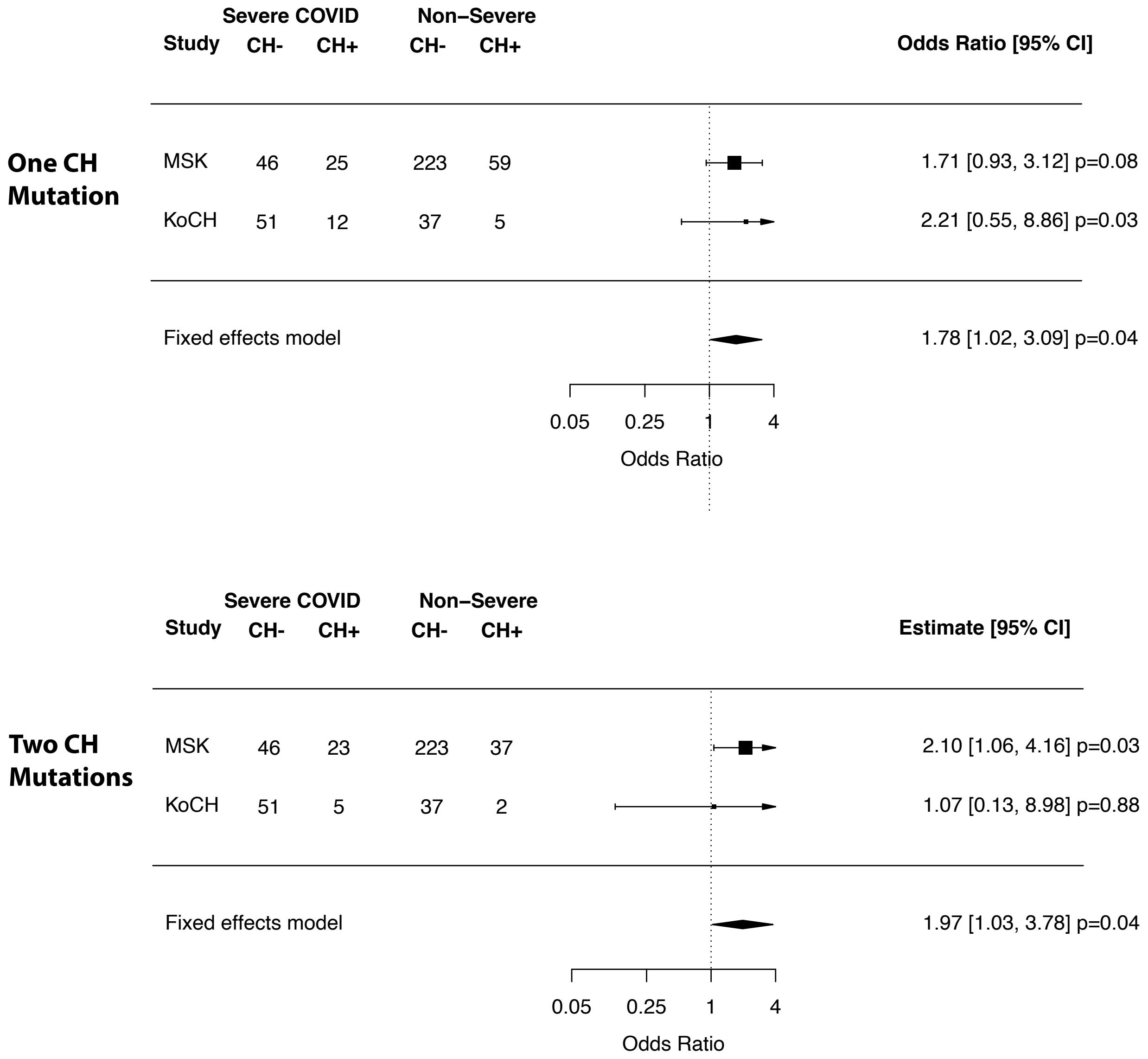

Supplementary Figure 4. Association between CH and Covid-19 severity stratified by the number of mutations. Shown are the odds ratio, 95% confidence interval and p-value (not adjusted for multiple hypothesis testing) from logistic regression adjusted for age, gender, race, smoking, diabetes, cardiovascular disease, COPD/asthma, BMI and month of Covid-19 diagnosis in 525 individuals. The MSK cohort was also adjusted for cancer primary site, exposure to cytotoxic cancer therapy before and after blood draw. Summary statistics for a fixed effects meta-analysis are shown. Any CH includes both driver (CH-PD) and non-driver mutations (CH-non-PD).

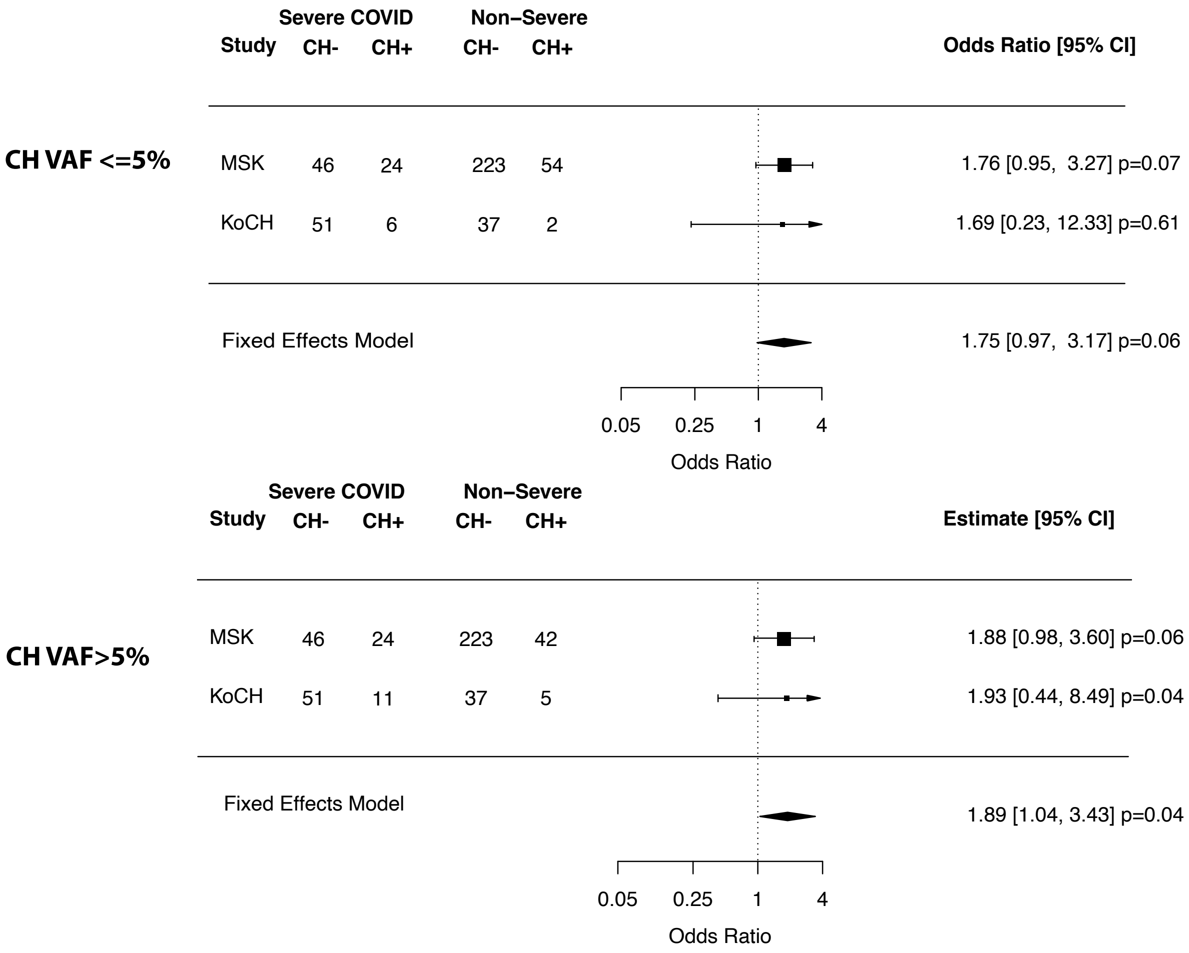

Supplementary Figure 5. Association between maximum VAF of CH-mutation(s) and Covid-19 severity. Shown are the odds ratio, 95% confidence interval and p-value (not adjusted for multiple hypothesis testing) from logistic regression adjusted for age, gender, race, smoking, diabetes, cardiovascular disease, COPD/asthma, BMI and month of Covid-19 diagnosis in 525 individuals. The MSK cohort was also adjusted for cancer primary site, exposure to cytotoxic cancer therapy before and after blood draw. Summary statistics for a fixed effects meta-analysis are shown. Any CH includes both driver (CH-PD) and non-driver mutations (CH-non-PD).

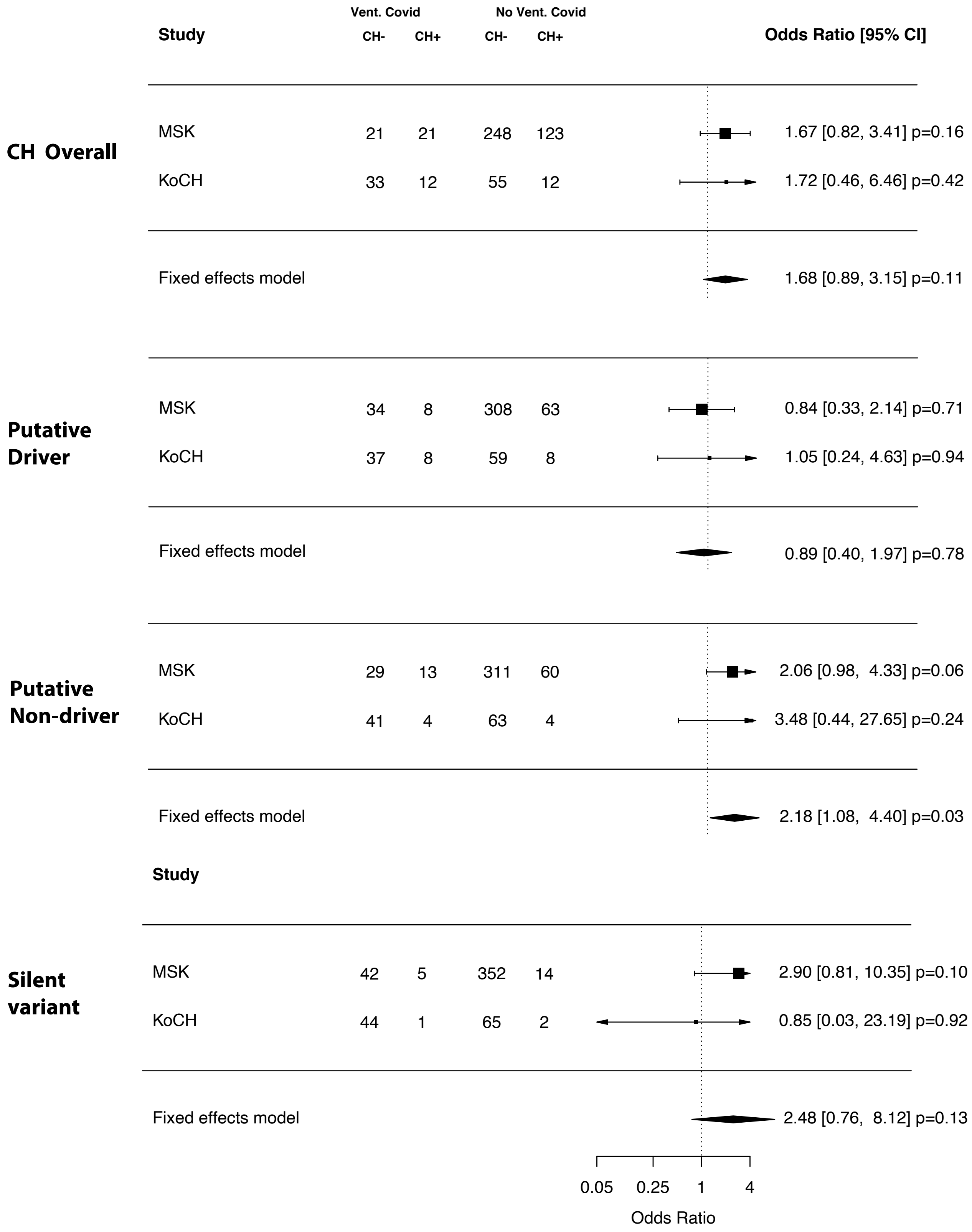

Supplementary Figure 6. Association between CH and non-invasive/invasive ventilation related to Covid-19. Shown are the odds ratio, 95% confidence interval and p-value (not adjusted for multiple hypothesis testing) from logistic regression adjusted for age, gender, race, smoking, diabetes, cardiovascular disease and COPD/asthma in 525 individuals. The MSK cohort was also adjusted for cancer primary site, exposure to cytotoxic cancer therapy before and after blood draw. Summary statistics for a fixed effects meta-analysis are shown. CH overall includes both driver and non-driver mutations.

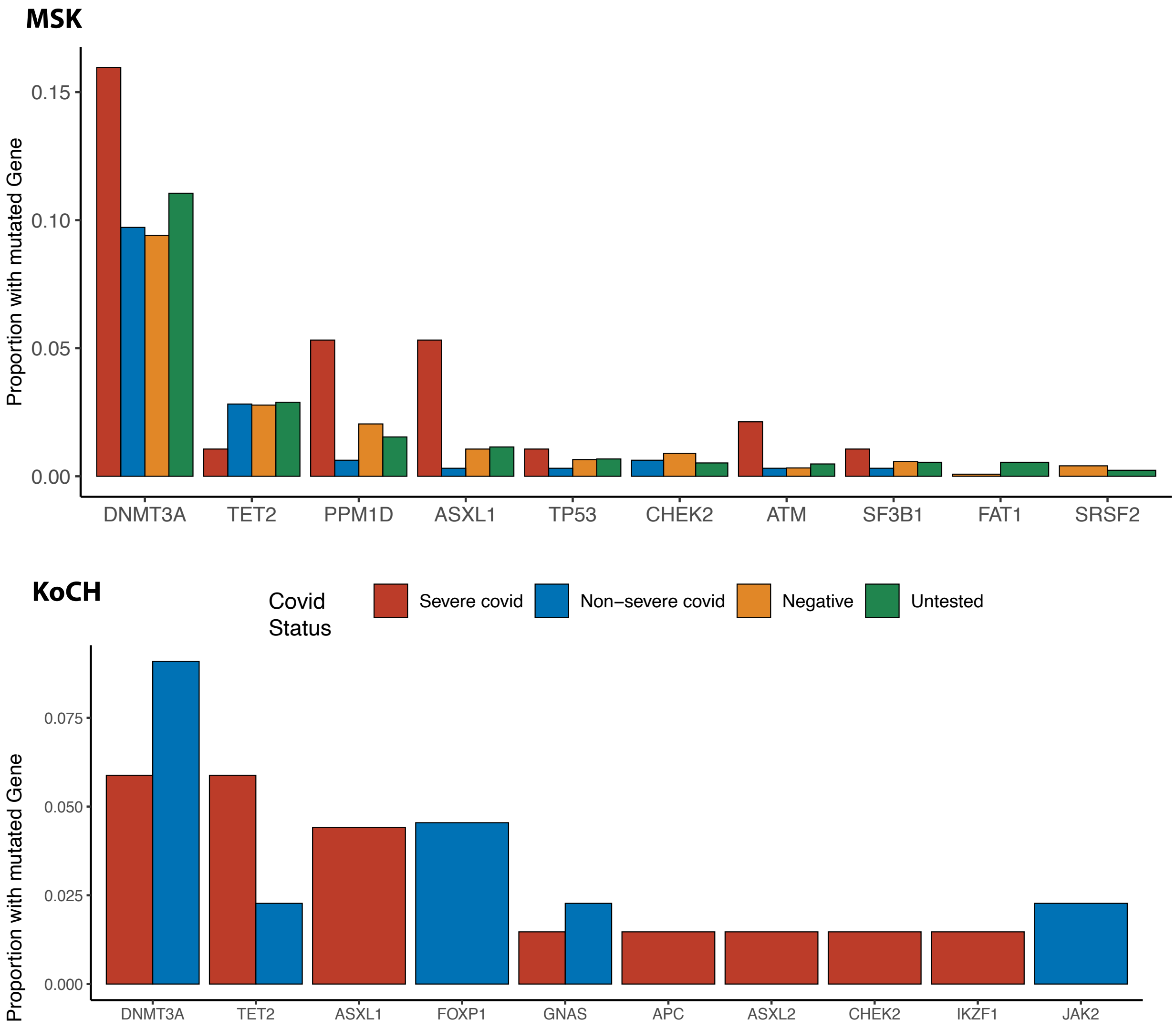

Supplementary Figure 7. Frequency of CH mutation by genes and Covid-19 infection and severity status. Shown are both CH-PD and CH-non-PD mutations.

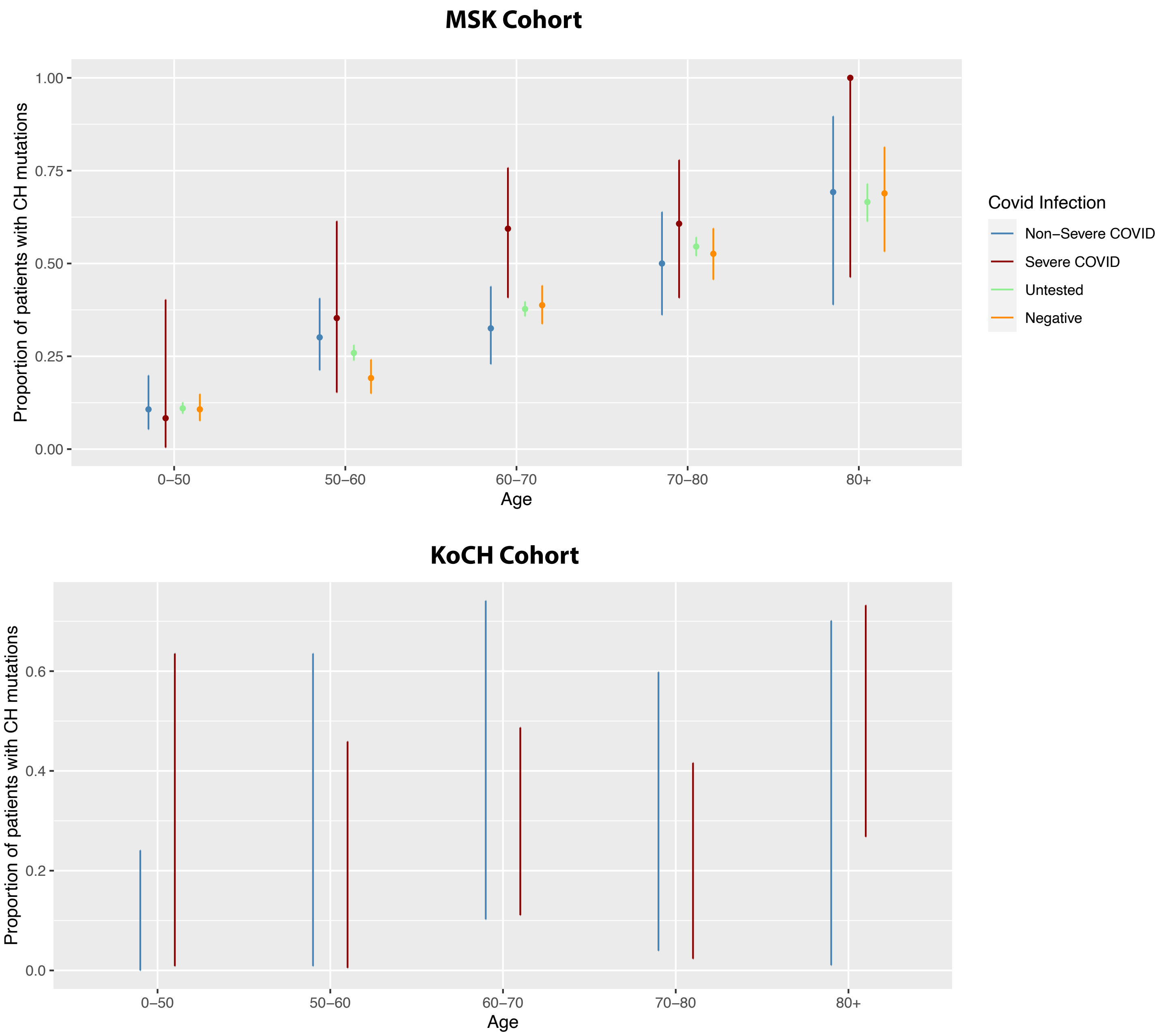

Supplementary Figure 8. Proportion of individuals with CH by Covid-19 severity, testing status and age group. CH is defined here as any mutation including both driver (CH-PD) and non-driver mutations (CH-non-PD). Error bars represent the 95% CI for the proportion based on a two-sided binomial test. The MSK cohort included 94 individuals with severe Covid-19, 319 with non-severe Covid-19, 1223 individuals who were tested for Covid-19 but found to be negative (Negative) and 7681 individuals who did not receive testing for Covid-19 during the follow-up period (Untested). The KoCH cohort included 68 individuals with severe Covid-19 and 44 individuals with non-severe Covid-19 infections.

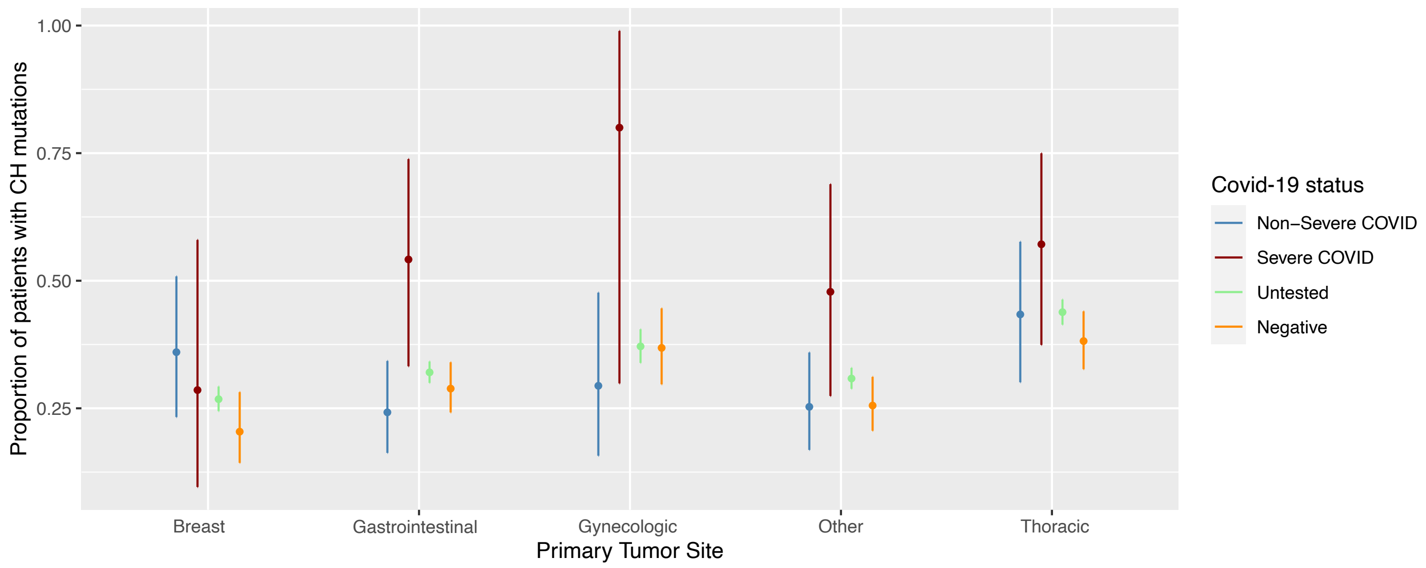

Supplementary Figure 9. Proportion of individuals with CH by Covid-19 severity and the most common primary tumor sites. CH is defined here as any mutation including both driver (CH-PD) and non-driver mutations (CH-non-PD). Error bars represent the 95% CI for the proportion based on a two-sided binomial test. Included are 1808 individuals with breast cancer, 4243 with gastrointestinal cancer, 1436 with gynecologic cancers, 3241 with thoracic cancers and 3520 with another cancer type (Other).

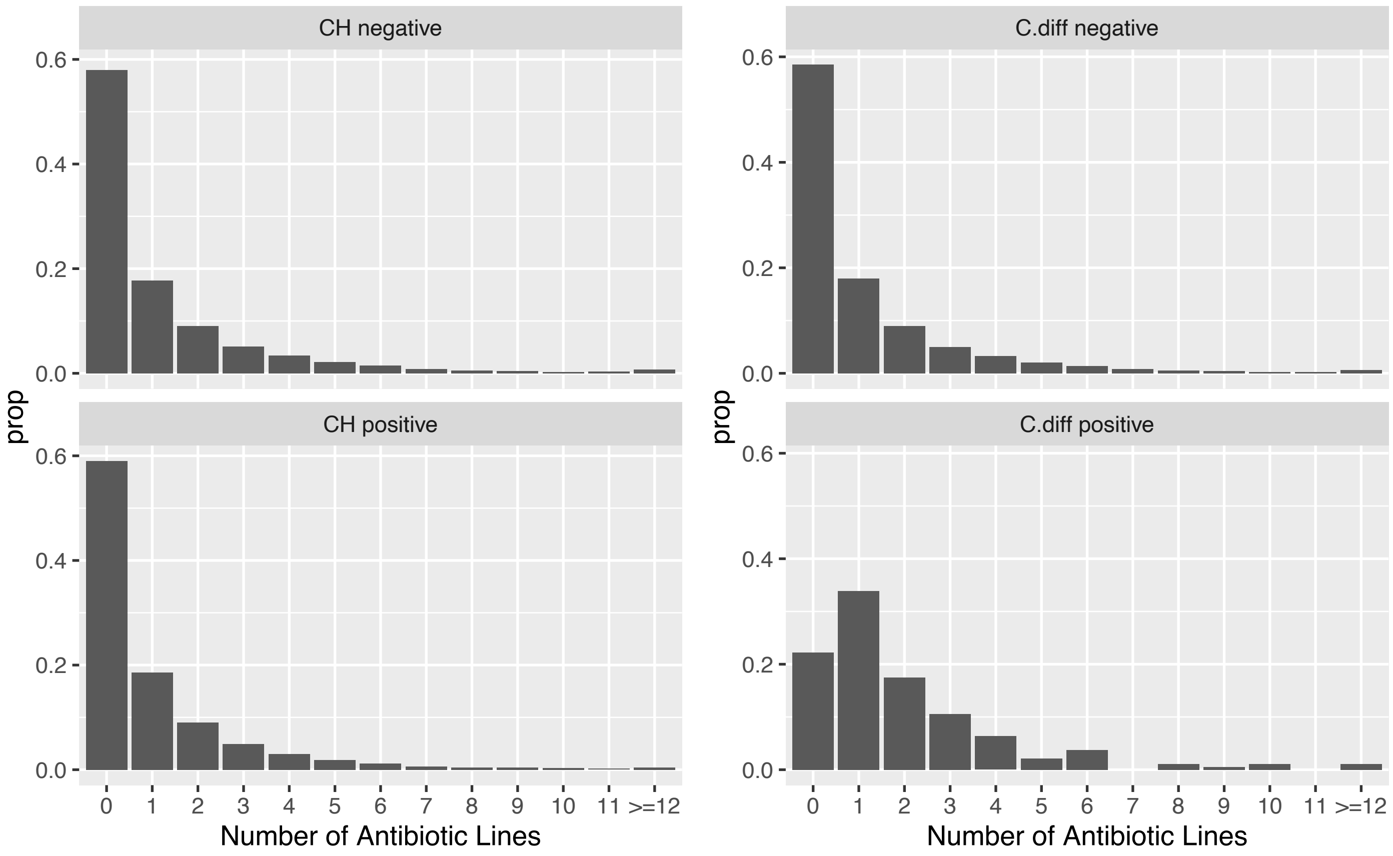

Supplementary Figure 10. Number of lines of antibiotics by the presence of clonal hematopoiesis and the development of clostridium difficile (C. diff) infection. We defined antibiotics lines as the number of unique antibiotic courses received at least two weeks apart where the start date was after IMPACT blood draw for CH profiling and before the date of last follow-up or Clostridium Difficile infection diagnosis whichever occurred first. CH is defined here as any mutation including both driver (CH-PD) and non-driver mutations (CH-non-PD).
