# Supplementary material for: Clonal hematopoiesis is associated with risk of severe Covid-19
Source: Nat Commun. 2021 Oct 13;12:5975. doi: 10.1038/s41467-021-26138-6 (PMC8514469; doi:10.1038/s41467-021-26138-6)
Supplement: Supplementary file 3 — Description of Additional Supplementary Files [file 41467_2021_26138_MOESM3_ESM.pdf]

Description of Additional Supplementary Files

Supplementary Data 1. List of genes included on IMPACT by first design version.
Supplementary Data 2. List of genes included on KoCH panel.
Supplementary Data 3. Hazard Ratios and associated statistics for CH and infection risk.
