# Supplementary material for: Clonal hematopoiesis is associated with risk of severe Covid-19
Source: Nat Commun. 2021 Oct 13;12:5975. doi: 10.1038/s41467-021-26138-6 (PMC8514469; doi:10.1038/s41467-021-26138-6)
Supplement: Supplementary file 7 — Reporting summary [file 41467_2021_26138_MOESM7_ESM.pdf]

nature research

Corresponding author(s): Kelly Bolton, Ahmet Zehir

Last updated by author(s): 2/10/2021

Reporting Summary

Nature Research wishes to improve the reproducibility of the work that we publish. This form provides structure for consistency and transparency in reporting. For further information on Nature Research policies, see Authors & Referees and the Editorial Policy Checklist .

Statistics

For all statistical analyses, confirm that the following items are present in the figure legend, table legend, main text, or Methods section.

n/a	Confirmed	
☐	☒	The exact sample size (*n*) for each experimental group/condition, given as a discrete number and unit of measurement
☐	☒	A statement on whether measurements were taken from distinct samples or whether the same sample was measured repeatedly
☐	☒	The statistical test(s) used AND whether they are one- or two-sided<br>*Only common tests should be described solely by name; describe more complex techniques in the Methods section.*
☐	☒	A description of all covariates tested
☐	☒	A description of any assumptions or corrections, such as tests of normality and adjustment for multiple comparisons
☐	☒	A full description of the statistical parameters including central tendency (e.g. means) or other basic estimates (e.g. regression coefficient) AND variation (e.g. standard deviation) or associated estimates of uncertainty (e.g. confidence intervals)
☐	☒	For null hypothesis testing, the test statistic (e.g. *F*, *t*, *r*) with confidence intervals, effect sizes, degrees of freedom and *P* value noted<br>*Give P values as exact values whenever suitable.*
☒	☐	For Bayesian analysis, information on the choice of priors and Markov chain Monte Carlo settings
☒	☐	For hierarchical and complex designs, identification of the appropriate level for tests and full reporting of outcomes
☒	☐	Estimates of effect sizes (e.g. Cohen's *d*, Pearson's *r*), indicating how they were calculated

Our web collection on statistics for biologists contains articles on many of the points above.

Software and code

Policy information about availability of computer code

Data collection	No software was used for the data collection
Data analysis	R version 4.0.1 was used to analyze the data in this study; R code used is available on GitHub. The following packages were used in our analyses. Sequencing data for the IMPACT study was analyzed using BWA (version 0.7.5a), ABRA (version 0.92), Genome Analysis Toolkit (GATK) (version 3.3-0), Mutect (version 1.1.4), VarDict (version 1.4.6), Somatic Indel Detector (version 2.3), VEP (version 86). Sequencing data for the KoCH cohort was analyzed using SeqPrep (version 0.3), Sickle (version 1.33), BWA-MEM (version 0.7.10), PICARD (version 1.94), GATK light (version 2.3.9), SNver (version 0.4.1), LoFreq (verison 0.6.1), GATK UnifiedGenotyper (version 2.3.9).

For manuscripts utilizing custom algorithms or software that are central to the research but not yet described in published literature, software must be made available to editors/reviewers. We strongly encourage code deposition in a community repository (e.g. GitHub). See the Nature Research guidelines for submitting code & software for further information.

Data

Policy information about availability of data

All manuscripts must include a data availability statement. This statement should provide the following information, where applicable:

- Accession codes, unique identifiers, or web links for publicly available datasets
- A list of figures that have associated raw data
- A description of any restrictions on data availability

The following data availability statement has been added "The minimal clinical and mutational data necessary to replicate the findings in the article are publicly available on Github: https://github.com/kbolton-lab/papers/tree/main/CH_COVID_NatureComm2021"

Field-specific reporting

Please select the one below that is the best fit for your research. If you are not sure, read the appropriate sections before making your selection.

☒ Life sciences ☐ Behavioural & social sciences ☐ Ecological, evolutionary & environmental sciences

For a reference copy of the document with all sections, see nature.com/documents/nr-reporting-summary-flat.pdf

Life sciences study design

All studies must disclose on these points even when the disclosure is negative.

Sample size	The sample size for MSK and KoCH was determined based on the number of individuals with Covid-19 and blood sequencing data available as of the time of the data freeze following the end of the first wave of Covid-19. Due to the unique circumstances of the pandemic, we chose to move forward with the analysis with our available sample size.
Data exclusions	For the IMPACT cohort, subjects were excluded who had an active hematologic malignancy at the time of blood draw. Seven subjects with Covid-19 had minimal documentation of clinical course following Covid-19 infection and were excluded. In three individuals with metastatic cancer there was clear progression of disease at the time of Covid-19 and it was unclear whether documented hypoxia could be attributed to Covid-19 or disease progression. These subjects were also excluded. For the KoCH cohort, Subjects who had an active malignancy at the time of blood draw were excluded.
Replication	Our main hypothesis and analysis was the association between clonal hematopoiesis (CH) and Covid-19 severity. We show through a combined analysis in two cohorts (MSK and KoCH) that CH is associated with Covid-19 severity. In secondary (exploratory) analyses we study the relationship between CH mutation types and Covid-19 severity and the relationship between CH and infection risk (in the MSK cohort). We present this manuscript alongside that of Zekavat et al. which also shows, using a different CH detection methodology and in a different population, that CH is associated with risk of severe Covid-19 and certain classes of infection. While not a exact replication due to differences in CH detection methodology and the definition of Covid-19 severity and infection classification, we view the findings of these two manuscripts as supporting our main conclusions that CH is associated with Covid-19 severity and infection risk.
Randomization	As this was not a randomized study, this was not relevant.
Blinding	Data collection took place independently of mutational analysis. Mutational analysis occurred prior to the Covid-19 outbreak and so data analysts were in effect blinded to Covid-19 outcomes. Clinical and mutational data frames were processed and analyzed separately before combining.

Reporting for specific materials, systems and methods

We require information from authors about some types of materials, experimental systems and methods used in many studies. Here, indicate whether each material, system or method listed is relevant to your study. If you are not sure if a list item applies to your research, read the appropriate section before selecting a response.

Materials & experimental systems

n/a	Involved in the study	
☒	☐	Antibodies
☒	☐	Eukaryotic cell lines
☒	☐	Palaeontology
☒	☐	Animals and other organisms
☐	☒	Human research participants
☐	☒	Clinical data

Methods

n/a	Involved in the study	
☒	☐	ChIP-seq
☒	☐	Flow cytometry
☒	☐	MRI-based neuroimaging

Human research participants

Policy information about studies involving human research participants

Population characteristics	The MSK cohort includes 9,307 patients with non-hematologic cancers treated at MSKCC who were alive on March 1st 2020 and who had their blood sequencing before this date using the MSK-IMPACT panel through an institutional prospective tumor sequencing protocol. Within the MSK cohort, 58% were female, 74% were of white non-hispanic race and the mean age was 59. Among 162 Covid-19 positive individuals, 94 had severe disease. 35% of individuals had clonal hematopoiesis.<br><br>The KoCH cohort includes laboratory confirmed patients with Covid-19 who were hospitalized between January and April 2020 in four tertiary hospitals in the Republic of Korea. Within the KoCH cohort, 46% were female, 100% were of east-asian race and the mean age was 63. 21% of inidivudals had clonal hematopoiesis
Recruitment	For the MSK cohort, consent to the MSK-IMPACT protocol occurred prior to Covid-19 infection. Patients sequenced on MSK-IMPACT are more likely to have advanced disease and more likely to have certain primary sites compared to the general solid tumor population in the U.S. However, since our study was focused on the association between Covid-19 and CH and since we did not observe evidence of heterogeneity in the association between Covid-19 and CH by tumor site , we do not anticipate the study population would bias our association results. For the KoCH cohort, patients were recruited serially between January and April of 2020 as they were admitted to four tertiary hospitals in the Republic of Korea. Patients with active malignancies were excluded. Since the KoCH cohort was obtained from hospitalized patients, a higher proportion of severe Covid-19 was observed than would be expected from the general population.
Ethics oversight	This study was approved by the Memorial Sloan Kettering IRB under protocol 12-245 part C and protocol 18-288. As stated in the manuscript, all patients enrolled on the MSK-IMPACT clinical protocol provided informed consent. A subset of patients that underwent tumor-genomic profiling as standard of care were not directly consented, in which case an IRB waiver was obtained to allow for inclusion into this study. The KoCH cohort included laboratory-confirmed patients with Covid-19 between January and April 2020 in four tertiary hospitals in Republic of Korea, Seoul National University Hospital, Seoul National University Bundang Hospital, National Medical Center and Kyungpook National University Hospital. The Seoul National University Hospital IRB, Seoul National University Bundang Hospital IRB, National Medical Center IRB and Kyungpook National University Hospital IRB approved the study (IRB numbers 2003-141-1110, B-2006/616-409, NMC-2008-050, KNUH-2020-04-069-001, respectively).

Note that full information on the approval of the study protocol must also be provided in the manuscript.

Clinical data

Policy information about clinical studies

All manuscripts should comply with the ICMJEguidelines for publication of clinical research and a completedCONSORT checklist must be included with all submissions.

Clinical trial registration	The MSK cohort is registered under NCT01775072.
Study protocol	Details available at ClinicalTrials.gov NCT01775072 or upon request.
Data collection	Data for the MSK IMPACT cohort was extracted through the electronic health record at Memorial Sloan Kettering Cancer Center. For the KoCH cohort, clinical and laboratory characteristics were retrospectively reviewed using the electronic health record of each institutions hospital. For the MSK-IMPACT cohort subjects were recruited from January 1st 2014 through July 1st 2020. Clinical data was obtained during the months of May 2020-August of 2020. For the KoCH cohort, subects were recruited between January and April 2020. Clinical data was obtained between 5/1/20-6/30/20.
Outcomes	The primary outcome was Covid-19 related hypoxia requiring supplemental oxygen (defined as supplemental oxygen device >1L with documented hypoxia or oxygen saturation of <94%) as documented in clinical records. As a secondary outcome for Covid-19 severity we used the need for invasive/non-invasive ventilation for hypoxia or oxygen saturation of <94%) as documented in clinical records.
